# The National Pension Fund

**Published:** 1888-10-13

**Authors:** 


### THE NATIONAL PENSION FUND.

THE first meeting of The Hospitals Association was held on Wednesday, the 10th inst., in the large hall of St. Thomas's Hospital, Dr. Bristowe, F.R.S., presiding. There was a good attendance of nurses and others interested.

A paper on the position and prospects of the National Pension Fund for Nurses was read by Mr. E. T. Clifford, hon. manager of the Fund. Mr. Clifford rapidly reviewed the early history of the Fund, calling attention to the fact that among other benefactors Mr. Burdett had paid the preliminary expenses, amounting to not less than £500. The attack made by the *Lancet* on the Fund some time ago was also noticed, and it was stated by Mr. Clifford that the publicity thus given had been of very great service. For the first few months after the commencement of business there arose such a pressure of affairs that the working staff had proved quite inadequate. Under these circumstances it had been found necessary to place the practical management in the hands of those trained in insurance affairs. The new administration, accustomed to deal with every variety of insurance business, had rapidly diminished the congestion. The first step after the business in hand had been dealt with was to issue a new and popular prospectus. The former prospectus had been prepared on the lines usually followed by ordinary insurance offices, and had proved too difficult for the intelligent comprehension of the average nurse. The new prospectus, which was not less complete as to its technical details, explained in popular language and with suitable illustrations the facts and principles on which annuities and pensions are based. Nurses all over the kingdom at once showed their appreciation of this effort to meet their needs. They now seemed for the first time to understand what the Fund was really capable of doing for them, and with rapid intuition began to take extensive advantage of its proposals. Many thousands of letters and inquiries had been received, and about five hundred proposals had been made and completed. A sum of £3,500 had been received from nurses in premiums alone. Mr. Clifford's address proved a great success, and was frequently applauded.

Dr. Bristowe, Mr. Burdett, Mr. George King, Mr. Thomas Ryan, Dr. Potter, and others, joined in the discussion which followed. It was pointed out that the issue of five hundred policies within a few months of the establishment of a Fund like this, was unprecedented in the history of insurance companies, and proved the genuine success the National Pension Fund undoubtedly was. Proposals were being accepted at the rate of five per diem on an average, and the honour of being amongst the first thousand nurses who joined, and who would be handed down as the original founders of the Fund, was being keenly competed for. Mr. Ryan stated that the nurses did not appear to be alive to the fact that hospital committees were waiting for the nurses on their staffs to send in a memorial expressing their desire to join, when the committees would be prepared to help their nurses by paying part of the premiums. At least this was the case at St. Mary's Hospital, where the nurses, for some reason or other, had failed to apply to the governors, though the latter were ready to help them materially when they took this step. Nurses should join at once by paying one shilling a month under the tables in the new prospectus, and then decide at their leisure how much more they could afford to pay in. The great thing was to become members of the Fund so as to protect themselves in case of illness or misfortune. Several delegates were present from provincial hospitals, Lincoln and Gloucester being especially well represented, but, though a misunderstanding, they did not speak. We propose to publish a full report next week, and shall be glad to include any remarks by delegates, providing they forward them to 38, Old Jewry, E.C., on or before Tuesday next.

